# Amber Light (590 nm) Induces the Breakdown of Lipid Droplets through Autophagy-Related Lysosomal Degradation in Differentiated Adipocytes

**DOI:** 10.1038/srep28476

**Published:** 2016-06-27

**Authors:** Min Sik Choi, Hyoung-June Kim, Mira Ham, Dong-Hwa Choi, Tae Ryong Lee, Dong Wook Shin

**Affiliations:** 1 Basic Research & Innovation Division, Amorepacific Corporation R&D Center, Yongin-si, Gyeonggi-do, 446–729, Republic of Korea; 2 Gyeonggi Bio Center, Gyeonggi Institute of Science & Technology Promotion, Suwon, Republic of Korea

## Abstract

Lipolysis in the adipocytes provides free fatty acids for other tissues in response to the energy demand. With the rapid increase in obesity-related diseases, finding novel stimuli or mechanisms that regulate lipid metabolism becomes important. We examined the effects of visible light (410, 457, 505, 530, 590, and 660 nm) irradiation on lipolysis regulation in adipocytes differentiated from human adipose-derived stem cells (ADSCs). Interestingly, 590 nm (amber) light irradiation significantly reduced the concentration of lipid droplets (LDs). We further investigated the lipolytic signaling pathways that are involved in 590 nm light irradiation-induced breakdown of LDs. Immunoblot analysis revealed that 590 nm light irradiation-induced phosphorylation of hormone-sensitive lipase (HSL) was insufficient to promote reduction of LDs. We observed that 590 nm light irradiation decreased the expression of perilipin 1. We found that 590 nm light irradiation, but not 505 nm, induced conversion of LC3 I to LC3 II, a representative autophagic marker. We further demonstrated that the lysosomal inhibitors leupeptin/NH_4_Cl inhibited 590 nm light irradiation-induced reduction of LDs in differentiated adipocytes. Our data suggest that 590 nm light irradiation-induced LD breakdown is partially mediated by autophagy-related lysosomal degradation, and can be applied in clinical settings to reduce obesity.

As the main reservoir of the body’s fuel, white adipose tissues play a critical role in energy storage and balance. During fasting or exercise, mature adipocytes provide energy as free fatty acids for other tissues through a process known as lipolysis^1^. Decreased lipolysis activity in adipose tissues may lead to triglyceride (TG) accumulation in lipid droplets (LDs), causing unnecessary additional energy intake, which leads to obesity^2^,^3^. Thus, tight control of lipolysis is required not only for energy homeostasis, but also for the prevention of metabolic diseases.

Lipolysis is a series of processes involving several regulatory events^4^. These include the lipolytic and anti-lipolytic mechanisms induced by lipolytic hormones such as adrenocorticotropic hormone (ACTH), anti-lipolytic hormones such as insulin, or LD-associated proteins such as perilipins and lipases^4^,^5^. Perilipin 1 has two modes of action: downregulating basal lipolysis and mediating protein kinase A (PKA)-activated lipolysis. Perilipin 1 is highly phosphorylated by cyclic AMP-dependent PKA^6^. A study showed that basal lipolysis is increased and PKA-stimulated lipolysis increase is blocked in adipocytes of perilipin 1 knock-out mice^7^,^8^.

Lipases play an important role in lipolysis. Even though there are many proteins predicted to have lipase or esterase activity in adipose tissues, hormone-sensitive lipase (HSL) and adipose triglyceride lipase (ATGL) are responsible for most of the triacylglycerol (TAG) hydrolase activity in murine adipocytes^9^. Several papers have reported that ATGL is the rate-limiting enzyme for the initiation of PKA-stimulated lipolysis and predominantly responsible for the first step of TAG hydrolysis, whereas HSL is primarily responsible for the hydrolysis of diacylglycerol (DAG)^10^,^11^,^12^. Other groups, however, have suggested that ATGL is less important in human adipocytes and that HSL fulfills the function as the rate-limiting TAG hydrolase in human adipocytes^13^,^14^.

There are several reports on the effects of several visible lights on various cells. Blue light exposure decreased proliferation of keratinocytes^15^,^16^. Red light exposure induces increased proliferation in keratinocytes^17^ and fibroblasts^18^. In addition, some visible lights are involved in the regulation of barrier recovery. Red light (550~670 nm) can accelerate recovery in a disrupted skin model, whereas blue light (430–510 nm) retards this recovery^19^. We also reported that violet light reduces the expression of keratinocyte differentiation markers through rhodopsin^20^.

In the present study, we examined the effects of visible light irradiation on adipocytes differentiated from human adipose-derived stem cells (ADSCs). We found that 590 nm light irradiation induced the breakdown of LDs in adipocytes, and further examined the underlying mechanism to explain the phenomenon.

## Results

### Visible light irradiation at 590 nm reduced LDs in differentiated adipocytes

**Figure 1:**
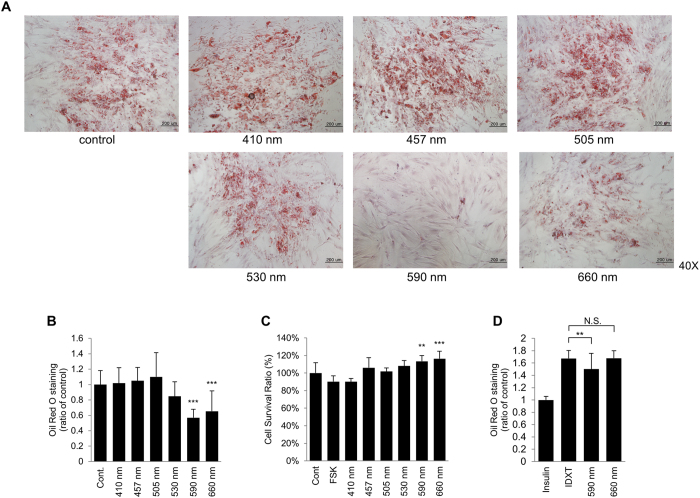
Effect of visible light irradiation on lipolysis in differentiated adipocytes. Differentiated adipocytes were irradiated with each visible light (410 nm, 457 nm, 505 nm, 530 nm, 590 nm and 660 nm) at dose of 18 J/cm^2^ 5 times (once a day, for 5 days). LDs in adipocytes were stained with Oil Red O dye (**a)** and quantified (***p < 0.001, ANOVA followed by Dunnett’s post hoc test; n = 22) (**b**). Cell viability after light exposure was evaluated using the CCK-8 assay (**p < 0.01, ***p < 0.001, ANOVA followed by Bonferroni post hoc test; n = 12) (**c**). Adipocytes were irradiated with 590 nm or 660 nm of visible light every 3 days during the differentiation process until 6 days. IDXT was used for positive control (**p < 0.01, ANOVA followed by Dunnett’s post hoc test; n = 18) (**d**). Data were normalized by setting the control as 1 (**b,d**) or 100% (**c**) and presented as means ± S.D. Scale bar = 200 μm. N.S. = not significant (p > 0.05).

To investigate the effects of visible light irradiation (410, 457, 505, 530, 590, or 660 nm) on fully differentiated adipocytes (2 weeks), cells were irradiated with each wavelength 5 times (once a day, for 5 days, which showed the maximum effects), and LDs in adipocytes were measured by performing Oil Red O staining analysis. Interestingly, 590 nm light irradiation significantly reduced LDs by about 45% compared with control (Fig. 1a). Although 660 nm light irradiation also enhanced the breakdown of LDs by 40%, its effect was less potent than that of 590 nm light irradiation (Fig. 1a,b). We confirmed that 590 nm light irradiation had no cytotoxic effect on adipocyte survival by performing CCK-8 assay (Fig. 1c). In addition, we examined whether 590 nm light irradiation has any effect on adipocyte differentiation. Cells were irradiated with 590 nm visible light every 3 days (day 0, 3, and 6) during the differentiation process. Oil red O staining analysis revealed that 590 nm visible light show slight decrease of LDs in 6 days during adipocyte differentiation (Fig. 1d). In addition, we confirmed that 590 nm light irradiation reduced LDs in a time-dependent manner (Fig. S1). These results indicated that 590 nm light irradiation most potently induced the breakdown of LDs in differentiated adipocytes.

### Irradiation at 590 nm did not affect the phosphorylation level of HSL

**Figure 2:**
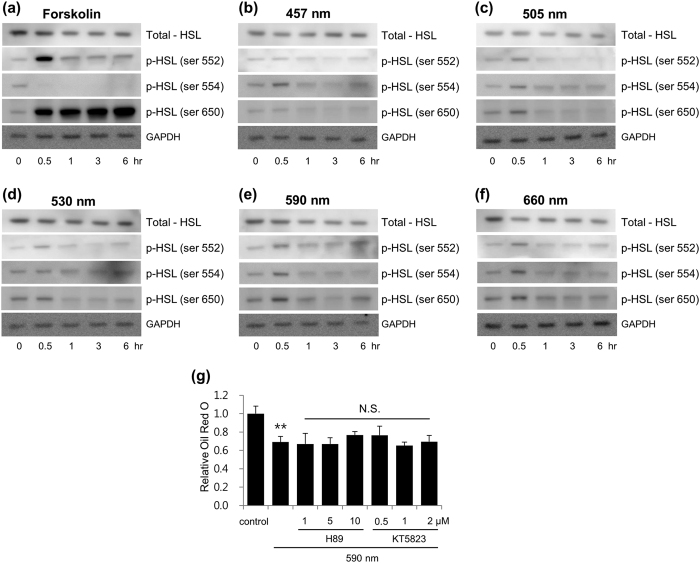
Phosphorylation of HSL in differentiated adipocytes irradiated with visible light. Differentiated adipocytes were incubated with forskolin (**a**) or irradiated with 457 nm (**b**), 505 nm (**c**), 530 nm (**d**), 590 nm (**e**), and 660 nm (**f**) of visible light (18 J/cm^2^) once for 30 min, and were supplemented with fresh media. Cell lysates were collected at the indicated time points (0, 0.5, 1, 3, 6 hr) and subjected to immunoblot for total HSL, phospho HSL (Ser552, Ser554 and Ser650), and GAPDH. These images are representative of three independent experiments. Differentiated adipocytes were irradiated with 590 nm light 3 times (18 J/cm^2^ a day, for 3 days) in the presence of H89 (1, 5 or 10 μM) or KT5823 (0.5, 1, 2 μM) at each concentration. LDs in adipocytes were stained with Oil Red O dye and quantified (**g**). **p < 0.01, compared to control by t-test. N.S., compared to 590 nm light irradiation-only group. ANOVA followed by Dunnett’s post hoc test.

For a better understanding of the mechanism involved in 590 nm light irradiation-induced decrease of LDs, we evaluated the effect of the irradiation on HSL, a key lipase regulated by reversible phosphorylation^9^,^11^,^14^. Our data revealed that 590 nm irradiation leads to a slight increase in HSL phosphorylation compared to other light wavelengths (Fig. 2b–e). However, comparing with the pattern of HSL phosphorylation by forskolin (Fig. 2a), the induction level or the duration of HSL phosphorylation by 590 nm irradiation was remarkably low or short. In addition, treatment with either H-89, a PKA inhibitor, or KT5823, a PKG inhibitor, did not affect 590 nm irradiation-induced breakdown of LDs (Fig. 2g).

### Irradiation at 590 nm specifically reduced the expression level of perilipin 1

**Figure 3:**
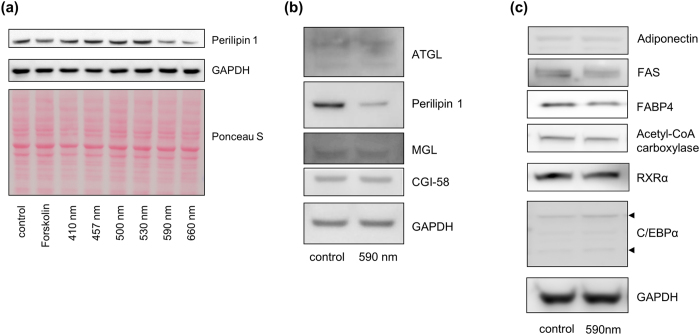
Effect of visible light irradiation on perilipin 1 expression in differentiated adipocytes. Differentiated adipocytes were incubated with forskolin or irradiated with 457 nm, 505 nm, 530 nm, 590 nm and 660 nm of visible light (18 J/cm^2^) 3 times (once a day, for 3 days). Cell lysates were collected and subjected to immunoblot for perilipin and GAPDH. Protein-transferred membrane was stained by Ponceau S staining before immunoblotting (**a**). Differentiated adipocytes were irradiated with 590 nm of visible light three times (18 J/cm^2^), and after 24 hr in fresh media, the cell lysates were collected and subjected to immunoblot for LD markers; ATGL, perilipin, MGL, and CGI-58 (**b**) and for adipogenic markers; adiponectin, FAS, FABP4, acetyl-CoA carboxylase, RXRα, C/EBPα (**c**). GAPDH was used as a loading control. These images were representative of three independent experiments.

In response to lipolytic stimulation by forskolin or β-adrenergic agents, cAMP-dependent PKA-mediated phosphorylation of perilipin 1 facilitates lipolysis-inducing HSL phosphorylation^11^,^21^. However, in the absence of lipolytic stimuli, namely, in basal or constitutive lipolysis, perilipin 1 negatively regulates lipase function^22^,^23^. Recently, it has been reported that a decrease in perilipin 1 protein expression could cause lipolysis under basal conditions^24^. Thus, we examined if perilipin 1 expression was affected by visible light irradiation. Interestingly, the expression level of perilipin 1 was significantly decreased by either 590 or 660 nm light irradiation (Fig. 3a), which was consistent with Oil-Red O staining results, as shown in Fig. 1. According to previous reports, forskolin increases the phosphorylation of perilipin 1 through cAMP-PKA signaling pathway^5^,^6^. Forskolin is not likely to affect perilipin 1 protein expression or degradation in adipocytes^25^. We also demonstrated that forskolin did not alter perilipin 1 expression compared to GAPDH expression (Fig. 3a).

The mass of adipose tissue can be reduced by increasing lipolysis, inhibiting adipogenesis or both^26^. Since 590 nm light irradiation decreased LD levels, we examined the expression levels of ATGL, monoacylglycerol lipase (MGL), and comparative gene identification-58 (CGI-58) that reside in LDs. We found that there were no changes in the expression level of ATGL, MGL, or CGI-58, despite a reduction in perilipin 1 (Fig. 3b). We further examined whether adipogenic markers were affected by 590 nm light irradiation in differentiated adipocytes. Although we could not find any difference in protein expression of adiponectin, acetyl-CoA carboxylase, retinoid X receptor α (RXR α) and CCAAT/enhancer-binding protein α (C/EBPα), we observed a slight reduction in both fatty acid synthase (FAS) and fatty acid binding protein 4 (FABP4) expression in the 590 nm light irradiated adipocytes (Fig. 3c). Next, we questioned whether the slight decrease of FAS expression could cause the inhibition of de novo lipogenesis resulting in the breakdown of LDs. It has been reported that the inhibition of FAS activity decreases LDs in adipocytes^27^. Thus, we tested whether 590 nm irradiation could change the FAS activity. We found that FAS activity in differentiated adipocytes after 590 nm light irradiation was slightly but not significantly decreased (Fig. S2), implying that 590 nm light irradiation-induced the breakdown of LDs are mainly due to lipolysis rather than the inhibition of *de novo* lipogenesis.

To further investigate whether lipid metabolism was altered by increased lipolysis, we examined the expression patterns of genes related to energy metabolism in irradiated adipocytes by qRT-PCR analysis^28^. Increases in expression of genes related to lipid metabolism such as acyl-CoA dehydrogenase medium chain (*ACADM*), acyl-CoA oxidase 1 (*ACOX1*) and carnitine palmitoyl transferase 1a/1b (*CPT1A, CPT1B*) were observed at 590 nm or 660 nm irradiated cells (Fig. S3).

### Irradiation at 590 nm enhanced the breakdown of LDs through autophagy-related lysosomal degradation

**Figure 4:**
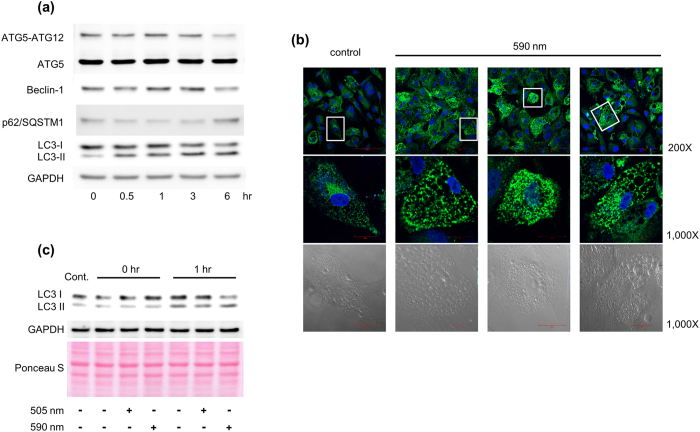
590 nm irradiation induced the conversion of LC3 I to LC3 II. Differentiated adipocytes were harvested at the indicated time points (0, 0.5, 1, 3, 6 hr) after 590 nm irradiation (18 J/cm^2^). The cell lysates were subjected to immunoblot for beclin-1, Atg5-Atg12 conjugate, p62/SQSTM1, LC3, and GAPDH (**a**). Differentiated adipocytes were fixed for immunofluorescence at 3 hr after 590 nm irradiation. LC3 puncta (green) was determined with anti-LC3 II antibodies under the confocal microscope (Zeiss LSM 700). DAPI (blue) was used to stain the nucleus of the cell. White box indicates position of high magnification images shown below (magnification 200X and 1,000X, Scale bar = 100 μm and 20 μm, respectively) (**b**). Differentiated adipocytes were harvested at each indicated time point (0, 1 hr) after 505 nm or 590 nm irradiation (18 J/cm^2^). The cell lysates were subjected to immunoblot for LC3 and GAPDH. Protein-transferred membrane was stained by Ponceau S staining before immunoblotting (**c**). These images were representative of three independent experiments.

Singh *et al*. suggested that LDs and autophagy components are interrelated^29^. Thus, to investigate whether the effects of 590 nm irradiation on the breakdown of LDs is mediated by autophagy pathway in differentiated adipocytes, we first examined the protein levels of beclin-1, Atg5-Atg12 conjugate, p62/SQSTM1, and LC-II, which are representative autophagy markers^29^. We found that the protein levels of beclin-1, Atg5-Atg12 conjugate, and LC3-II were increased after 590 nm light irradiation, whereas the protein level of p62/SQSTM1 was reduced (Fig. 4a). During the autophagy process, LC3-II is also characterized by a punctate pattern. We confirmed that LC3-II puncta was markedly increased compared with control at 3 hr after 590 nm light irradiation (Fig. 4b).

**Figure 5:**
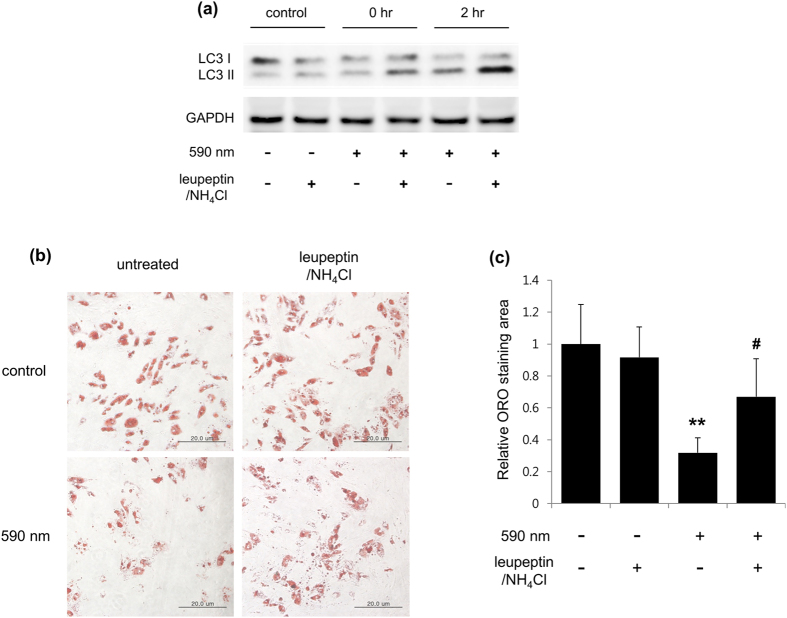
Leupeptin/NH_4_Cl blocked 590 nm–induced breakdown of LDs in differentiated adipocytes. Differentiated adipocytes were pretreated with or without both NH_4_Cl (20 mM) and leupeptin (Leu; 100 μM) and then were irradiated with 590 nm (18 J/cm^2^) once (**a**) or 3 times (once a day, for 3 days) (**b,c**). After 2 hr recovery following irradiation, cell lysates were analyzed by immunoblotting with antibodies against LC3 and GAPDH (**a**). After third irradiation of 590 nm, LDs in adipocytes were stained with Oil Red O dye (**b)** and quantified (**c**). These images were representative of three independent experiments. Scale bar = 20 μm. **p < 0.01, ANOVA followed by Bonferroni post hoc test (n = 4) was performed on the data of 3 groups (control, inhibitors-only group, and 590 nm irradiation-only group). ^#^p < 0.05, compared to 590 nm irradiation-only group by t-test.

To examine whether 590 nm specifically shows this phenomenon, we compared 590 nm light irradiation with 505 nm light irradiation. Interestingly, 590 nm light irradiation but not 505 nm enhanced the LC3-II levels at 1 hr post-irradiation (Fig. 4c). This increase in the LC3-II level could be due to an increased autophagosome formation or impaired autophagosome-lysosome fusion (autophagosome maturation)^30^. To discriminate between these two possibilities, we measured the level of LC3-II in the presence of both NH_4_Cl and leupeptin, which are used as a lysosome-neutralizing agent and a lysosomal proteolysis inhibitor, respectively^30^. Light irradiation at 590 nm in the presence of both NH_4_Cl and leupeptin increased the level of LC3-II in differentiated adipocytes (Fig. 5a), indicating that the increased LC3-II expression was due to increased autophagosome formation. In addition, 590 nm light irradiation-induced reduction of LDs was blocked in the presence of both NH_4_Cl and leupeptin (Fig. 5b,c).

## Discussion

In the present study, we showed that 590 nm irradiation significantly reduced LD levels in differentiated adipocytes (Fig. 1).

To elucidate the underlying mechanism, we examined the expression or phosphorylation status of photoreceptors, HSL, and perilipin 1. To find out the photoreceptor that responds to 590 nm visible light, we analyzed the membrane fraction of differentiated adipocytes on each day (0, 3, 7 days) using liquid-chromatography-tandem mass spectrometry, but we unfortunately could not observe any photoreceptor (Fig. S4).

According to Nilsson *et al*., if the ‘surge’ of HSL phosphorylation level falls sharply, the subsequent lipolysis can also shut down immediately^31^. This report implies that HSL phosphorylation induced by 590 nm irradiation could be too low and short to induce lipolysis sufficiently. Additionally, we confirmed that 590 nm irradiation-induced reduction of LDs was not affected by a PKA inhibitor or a PKG inhibitor, indicating that HSL was not significantly involved with adipocyte lipolysis by 590 nm irradiation.

Perilipins and lipases such as ATGL and its co-lipase CGI-58 are responsible for fat mobilization^26^. Intriguingly, 590 nm irradiation specifically reduced the expression level of perilipin 1 (Fig. 3), which indicates the contribution of increased lipolysis^7^,^8^. Meanwhile, patients with loss-of-function mutations in perilipin 1 exhibit elevated basal lipolysis. This led to severe metabolic consequences including partial lipodystrophy, severe insulin resistance, diabetes, dyslipidemia, and nonalcoholic fatty liver disease^32^,^33^, implying that reduced perilipin 1 level by 590 nm could be deleterious for normal metabolism. However, there are two differences between these previous data and our present study: (1) Lipolytic condition at 590 nm irradiation is a temporally acute, but not a ‘basal’ condition caused by continuous effects like a loss of function. (2) Though we found similar results with those of previous studies showing the reduction of LDs following the decreased expression level of perilipin 1, the significance of our data is limited only to local areas irradiated by 590 nm visible light. Consequently, the 590 nm irradiation employed in this study is more likely to induce the breakdown of LDs only in a particular area, rather than affect whole body metabolism by, for example, loss-of-function mutations in perilipin 1.

We found that 590 nm irradiation significantly reduced perilipin 1 protein levels, but not mRNA levels (Fig. 3 and Table S1). It is interesting to note that the reduction in perilipin 1 protein caused by tumor necrosis factor α treatment is associated with reduced perilipin 1 mRNA levels, whereas nelfinavir, a human immunodeficiency virus protease inhibitor, reduces perilipin 1 protein level, but does not decrease perilipin 1 mRNA^24^,^34^. Mechanisms controlling stability of perilipin 1 protein in adipocytes are largely unknown but may be functionally important because changes in the protein content of perilipin 1 are not always associated with alterations in mRNA levels^16^,^35^,^36^. A report demonstrated that perilipin 1 degradation is mediated by a ubiquitination-proteasome pathway, which enables post-translational regulation of perilipin 1^37^. However, we could not observe induction of proteasome-dependent perilipin 1 degradation by 590 nm irradiation because cell death occurred in our experimental condition, which required continuous treatment of MG132, a proteasome inhibitor (data not shown). Therefore, we could not exclude the possibility that perilipin 1 may be degraded via the proteasome pathway after 590 nm irradiation.

Recent studies revealed that a link between LDs and lysosomes is related to the mobilization of intracellular lipid stores. Although cytoplasmic lipases are important for LD degradation in adipocytes, where HSL and ATGL double knock-out abolishes more than 90% of lipolytic activity^9^, a different type of LD degradation process has been identified, named lipophagy^29^. In this process, autophagosomes contribute to the delivery of LDs to lysosomes, leading to their degradation. Recently, Kaushik *et al*. suggested that elimination of perilipin 2 and perilipin 3 by chaperone-mediated autophagy, which selectively degrades cytosolic proteins containing KFERQ motif in lysosomes, facilitates access of the lipid core to the cytosolic lipase ATGL^38^. Another study revealed that autophagy genes (ATGs) and ATG-positive membranes are detected in LDs during lipolysis, suggesting that autophagosomes form on the surface of LDs^39^. Similarly, we demonstrated that 590 nm irradiation, but not 505 nm, specifically induced accumulation of LC3 II and reduction of LDs. This was inhibited by leupeptin/NH_4_Cl (Fig. 5). Thus, we assumed that perilipin 1 degradation and reduction of LDs by 590 nm irradiation may be due to lysosomal degradation through the activation of autophagic flux.

In our study, we found that adipocyte-specific proteins such as ATGL, CGI-58, and C/EBPα showed no changes in their expression levels after 590 nm irradiation (Fig. 3). This phenomenon was similarly identified in a previous study. Tansey *et al*. demonstrated that perilipin null mice exhibited a lean phenotype, but no changes in expression levels of C/EBPα and diglyceride acyltransferase (DGAT)^8^. These data indicate that the decrease of perilipin, which drives the breakdown of LDs, is likely to show no direct relevance with the expression levels of adipocyte specific proteins contributing to form LDs.

A recent study also showed that Atg5 transgenic mice exhibit leanness with loss of weight during the aging process, indicating that the amount of fat in adipocytes is significantly decreased^40^. By next-generation sequencing, we further demonstrated that 590 nm-irradiated adipocytes showed higher expression of Atg5 gene and down-regulation of the expression of p62 when compared to control adipocytes (Table 1).

In conclusion, we suggest that 590 nm irradiation enhances the reduction of LDs in differentiated adipocytes, potentially via autophagy-related lysosomal degradation of perilipin 1. Our scientific approach to explain how 590 nm visible light could induce the breakdown of LDs may shed light on a potential non-invasive treatment for local fat reduction.

## Materials and Methods

### Reagents and antibodies

Forskolin (F6886), NH_4_Cl (A9434) and leupeptin (L2884) were purchased from Sigma–Aldrich (St. Louis, MO, USA). H89 (#371962) and KT5823 (#420321) were from Calbiochem (San Diego, CA, USA). Anti-perilipin 1 (#9349), anti-HSL (#4107), anti-pHSL (Ser552)(#4139), anti-pHSL (Ser554)(#4137), anti-pHSL (Ser650)(#4126), anti-acetyl-CoA carboxylase (#3676), anti-adiponectin (#2789), anti-CCAAT/enhancer-binding protein α (C/EBPα) (#8178), anti-fatty acid binding protein 4 (FABP4) (#3544) and anti-fatty acid synthase (FAS) (#3180), anti-p62/SQSTM1 (#5114s) antibodies were from Cell Signaling Technology (Beverly, MA, USA). Anti-beclin-1 (NB110-87318), anti-Atg5 (NB110-53818), and anti-LC3 antibody (NB100-2220) was purchased from Novus Biologicals (Littleton, CO, USA). Anti-GAPDH (SC-25778) antibody was from Santa Cruz Biotechnology (Dallas, TX, USA).

### ADSC culture and differentiation

Human adipose derived stem cells (ADSCs) were purchased from Lonza (Walkersville, MD, USA). Confluent ADSCs were incubated in ADSC differentiation medium containing 1 μg/ml insulin, 1 μM dexamethasone, 0.5 mM IBMX (three chemicals are called as IDX), 1 μM troglitazone for accelerating adipocyte differentiation compared with IDX (called as IDXT), and 10% FBS. Culture medium was changed every 2 days to a fresh one. Insulin (I5500), IBMX (I5879), dexamethasone (D8893) and troglitazone (T2573) were purchased from Sigma–Aldrich (St. Louis, MO, USA).

### LED irradiation system

The LED irradiation system, which emits light with a narrow bandwidth inside a CO_2_ incubator, was purchased from HanaroTR (Suwon, Korea). Internal LED arrays, which emit violet light (380–420 nm, λ_max_: 410 nm), green light (450–560 nm, λ_max_: 505 nm), orange light (560–620 nm, λ_max_: 590 nm) and red light (620–690 nm, λ_max_: 660 nm), could be controlled from 10 mW/cm^2^ for 30 min at one time when the object was 50 mm from the LED arrays. These arrays were obtained from Roithner Lasertechnik GmbH (Wien, Austria). We used a spectroradiometer SPR-4001 (Luzchem, Ottawa, Canada) to determine the intensity of the irradiation.

### Light exposure and treatment with inhibitors

The cells were washed twice with PBS prior to irradiation and irradiated in PBS with the indicated exposure lights to avoid the side effects associated with photosensitization in the culture medium. To avoid any confounding effects from other factors (temperature, humidity and CO_2_), the LED arrays were placed inside the CO_2_ cell culture incubator during exposure. The cells were then irradiated with LED lights at 18 J/cm^2^. For the inhibition experiments, cells were pre-incubated for 1 hr with each inhibitor.

### Cell viability tests

Adipocyte viability after light exposure was evaluated using the Cell Counting Kit (CCK)-8 assay according to the manufacturer’s instructions (Dojindo Molecular Technologies. Inc, Kumamoto, Japan). The cell viability detecting reagent, WST-8 [2-(2-methoxy-4-nitrophenyl)-3-(4-nitrophenyl)-5-(2,4-disulfophenyl)-*2H*-tetrazolium, monosodium salt] was added to the cells in culture, and the cells were incubated for 2 hr in a humidified atmosphere. The absorbance at 450 nm was then measured, and the cell viability was expressed as the percentage of the absolute optical density of each sample relative to that of the control value.

### Western blot analysis

Adipocytes were lysed in RIPA cell lysis buffer containing a protease inhibitor and phosphatase inhibitor cocktail (Sigma-Aldrich, St Louis, MO, USA). The lysate was then subjected to centrifugation at 15,000 × *g* for 20 min, and the supernatant was used for the analysis. The protein concentration was determined using the Bradford method with bovine serum albumin as the standard. The proteins (40 μg/well) were loaded and fractionated using SDS-PAGE and transferred onto nitrocellulose membranes. The membranes were blocked with 5% non-fat skim milk in TBST (10 mM Tris-HCl [pH 8.0], 150 mM NaCl, 0.015% Tween-20) for 1 hr at room temperature and then probed overnight at 4 °C with primary antibodies. All of the blots were washed 3 times with TBST and then probed with horseradish peroxidase-conjugated goat anti-mouse or -rabbit IgG secondary antibodies (Bio-Rad, Hercules, CA, USA) at room temperature for 1 hr. The membranes were developed using ECL solution (Amersham Pharmacia Biotech, Piscataway, NJ, USA).

### Oil Red O staining

Adipocyte lipolysis was assessed using an Oil Red O stain (O0625, Sigma–Aldrich, St. Louis, MO, USA) as an indicator of intracellular lipid accumulation. After the irradiation-induced lipolysis, cells were washed twice with PBS, fixed with 10% formalin in PBS for 1 h, and then washed with 60% isopropanol, before being allowed to dry completely. Adipocytes were stained with 0.2% Oil Red O reagent for 10 min at room temperature, and washed with H_2_O four times. Each sample was eluted with 100% isopropanol for 10 min and absorbance was measured at 505 nm using a spectrophotometer. To visualize the nucleus, adipocytes were counterstained with hematoxylin reagent (H3136, Sigma–Aldrich, St. Louis, MO, USA) for 2 min and washed twice with H_2_O. The level of adipocyte differentiation was observed using an inverted phase-microscope.

### Immunofluorescence

Adipocytes were fixed with 10% formaldehyde for immunostaining for 10 min and permeabilized with 0.1% Triton X-100 for 10 min. The blocking and diluent solutions consisted of PBS-T containing 1% BSA (Sigma–Aldrich, St. Louis, MO, USA). The cells were blocked at room temperature for 30 min and incubated for overnight at 4 °C with LC3 II antibody followed by incubation with an Alexa 488 secondary antibody (Invitrogen, Carlsbad, CA, USA) for 60 min. DAPI was used for the nuclei staining (Sigma–Aldrich, St. Louis, MO, USA). PBS-T washes were performed at each step. These images were observed using a Carl Zeiss LSM700 inverted confocal microscope and the image acquisition software ZEN (Carl Zeiss, Germany).

### Next-generation sequencing and data analysis

In order to construct cDNA libraries with the TruSeq RNA library kit, 1ug of total RNA was used. The protocol consisted of polyA-selected RNA extraction, RNA fragmentation, random hexamer primed reverse transcription and 100 nucleotide paired-end sequencing by Illumina HiSeq2000. The libraries were quantified using qPCR according to the qPCR Quantification Protocol Guide and qualified using an Agilent Technologies 2100 Bioanalyzer. To estimate expression levels and to find alternative spliced transcripts, the RNA-Seq reads were mapped to the human genome using TopHat^41^, which is capable of reporting split-read alignments across splice junctions and determined using Cufflinks software^42^ in default options. The reference genome sequence (hg19, Genome Reference Consortium GRCh37) and annotation data were downloaded from the UCSC website (http://genome.uscs.edu). The transcript counts in isoform level and gene level were calculated, and the relative transcript abundances were measured in FPKM (Fragments Per Kilobase of exon per Million fragments mapped) using Cufflinks. Raw data were extracted as FPKM values for each gene for each sample by cufflinks software. Transcripts with at least one zeroed FPKM values across all samples were excluded. We added 1 with FPKM value to facilitate log2 transformation. Filtered data was transformed by logarithm and normalized by quantile method. Differentially expressed genes (DEG) were adjusted |fold change|> = 2 for each comparison pair. All NGS data analysis and visualization of differentially expressed genes was conducted using R 2.15.1 (www.r-project.org).

### Statistical analysis

Data were analyzed by one-way analysis of variance (ANOVA) followed by post hoc test and were presented as means ± S.D. of at least three independent experiments. *P*-value of less than 0.05 was considered statistically significant. All statistical analyses were performed using SPSS software (Chicago, IL, USA).

## Additional Information

**How to cite this article**: Choi, M. S. *et al*. Amber Light (590 nm) Induces the Breakdown of Lipid Droplets through Autophagy-Related Lysosomal Degradation in Differentiated Adipocytes. *Sci. Rep.* **6**, 28476; doi: 10.1038/srep28476 (2016).

## Supplementary Material

Supplementary Information

## Figures and Tables

**Table 1:** Analysis of autophagy-related genes in 590 nm light irradiated adipocytes by next generation sequencing.

Gene symbol	Gene name	Gene ID	Transcript (Accession No.)	Fold Changes
ULK1	unc-51 like autophagy activating kinase 1	8408	NM_003565	−1.102478
ULK2	unc-51 like autophagy activating kinase 2	9706	NM_014683	1.53279
			NM_001142610	1.721826
ATG2A	autophagy related 2A	23130	NM_015104	−1.590105
ATG2B	autophagy related 2B	55102	NM_018036	2.656969
ATG3	autophagy related 3	64422	NM_022488	2.097698
ATG4A	autophagy related 4A, cysteine peptidase	115201	NM_052936	1.11266
			NM_178270	1.085157
ATG4B	autophagy related 4B, cysteine peptidase	23192	NM_013325	−1.540281
			NM_178326	−1.164792
ATG4C	autophagy related 4C, cysteine peptidase	84938	NM_178221	1.216723
			NM_032852	1.849622
ATG4D	autophagy related 4D, cysteine peptidase	84971	NM_032885	−1.764885
			NR_104024	−1.471855
			NR_104025	−1.680783
			NM_001281504	−1.879178
ATG5	autophagy related 5	9474	NM_001286106	−1.101114
			NM_001286107	1.195802
			NM_001286108	1.000001
			NM_004849	2.890485
BECN1	beclin 1, autophagy related	8678	NM_003766	1.306998
MAP1LC3A	microtubule-associated protein 1 light chain 3 alpha	84557	NM_181509	−1.045318
			NM_032514	−1.496713
MAP1LC3B	microtubule-associated protein 1 light chain 3 beta	81631	NM_022818	1.861213
MAP1LC3B2	microtubule-associated protein 1 light chain 3 beta 2	643246	NM_001085481	−1.250139
MAP1LC3C	microtubule-associated protein 1 light chain 3 gamma	440738	NM_001004343	−1.055382
GABARAPL1	GABA(A) receptor-associated protein like 1	23710	NM_031412	1.092751
GABARAPL2	GABA(A) receptor-associated protein-like 2	11345	NM_007285	1.166983
GABARAP	GABA(A) receptor-associated protein	11337	NM_007278	−1.366561
ATG9A	autophagy related 9A	79065	NM_001077198	−1.25456
			NM_024085	−1.708516
			NR_104255	−1.090341
ATG9B	autophagy related 9B	285973	NR_073169	−1.092852
ATG10	autophagy related 10	83734	NM_031482	−1.413795
ATG12	autophagy related 12	9140	NM_004707	1.802323
			NM_001277783	−1.050214
			NR_033362	−1.003666
			NR_033363	1.457552
			NR_073603	1.165688
ATG13	autophagy related 13	9776	NM_001205119	−1.0001
			NM_001205121	−1.314789
			NM_001205122	1.217307
			NM_001205120	−1.696294
			NM_014741	−1.355076
ATG14	autophagy related 14	22863	NM_014924	1.154586
ATG16L1	autophagy related 16-like 1 (S. cerevisiae)	55054	NM_017974	−2.113102
			NM_001190267	1.138508
			NM_198890	1.167225
			NM_001190266	−1.29171
			NM_030803	−1.423362
ATG16L2	autophagy related 16-like 2 (S. cerevisiae)	89849	NM_033388	−1.297997
RB1CC1	RB1-inducible coiled-coil 1	9821	NM_014781	2.468446
			NM_001083617	1.273012
WIPI1	WD repeat domain, phosphoinositide interacting 1	55062	NM_017983	1.107066
WIPI2	WD repeat domain, phosphoinositide interacting 2	26100	NM_001033520	1.049023
			NM_001033519	−1.950824
			NM_001278299	−1.227057
			NM_015610	−1.631746
			NM_001033518	−1.882272
			NM_016003	−1.65438
SQSTM1 (p62)	sequestosome 1	8878	NM_001142299	−2.760694
			NM_003900	−1.229996
			NM_001142298	−1.360369
UVRAG	UV radiation resistance associated	7405	NM_003369	1.270423
KIAA0226	KIAA0226	9711	NM_001145642	−1.189882
PIK3R4	phosphoinositide-3-kinase, regulatory subunit 4	30849	NM_014602	2.58321
PIK3C3	phosphatidylinositol 3-kinase, catalytic subunit type 3	5289	NM_002647	3.038093
LAMP1	lysosomal-associated membrane protein 1	3916	NM_005561	1.039644
MTOR (mTORC1)	mechanistic target of rapamycin (serine/threonine kinase)	2475	NM_004958	1.294603
