# Supplementary material for: Amber Light (590 nm) Induces the Breakdown of Lipid Droplets through Autophagy-Related Lysosomal Degradation in Differentiated Adipocytes
Source: Sci Rep. 2016 Jun 27;6:28476. doi: 10.1038/srep28476 (PMC4921916; doi:10.1038/srep28476)
Supplement: Supplementary Information [file srep28476-s1.doc]

**Amber light (590 nm) induces the breakdown of lipid droplets**

**through autophagy-related lysosomal degradation in differentiated adipocytes.**

Min Sik Choi^1,#^, Hyoung-June Kim^1,#^, Mira Ham^1^, Dong-Hwa Choi^2^, Tae Ryong Lee^1^,*, Dong Wook Shin^1^,*

^1^Basic Research & Innovation Division, Amorepacific Corporation R&D Center, Yongin-si, Gyeonggi-do, Republic of Korea. 446-729, ^2^Gyeonggi Bio Center, Gyeonggi Institute of Science & Technology Promotion, Suwon, Republic of Korea.

**SUPPLEMENTARY MATERIAL**

**Materials and Methods**

**FAS activity assay**

The FAS activity assay was performed as previously described with minor modifications. In brief, cell lysates were mixed with acetyl-CoA and NADPH in 0.2 M potassium phosphate buffer, 0.4 mM EDTA (pH 7.0) and incubated at 30°C for 10 min. The enzymatic reaction was initiated by adding 20 μl of malonyl-CoA solution (0.2 mM), and the decrease in OD was measured every 1 min for 30 min via kinetic measurements obtained on a microplate reader set at 340 nm. Based on the results, the overall FAS enzyme activity was estimated by calculating the NADPH oxidation, using ε = 6220 M^-1^cm^-1^. Acetyl-CoA (A2056), NADPH (N1630), and malonyl-CoA (M4263) were purchased from Sigma–Aldrich (St. Louis, MO, USA).

#### **RNA extraction and quantitative real-time RT-PCR (Q-RT-PCR)**

Total RNA was isolated using TRIzol™ (Invitrogen, Carlsbad, CA, USA), according to the manufacturer’s instructions. The RNA concentration was determined spectrophotometrically, and the integrity of the RNA was assessed using a BioAnalyzer 2100 (Agilent Technologies, Santa Clara, CA, USA). Two micrograms of RNA were reverse-transcribed into cDNA using SuperScript®III reverse transcriptase (Invitrogen, Carlsbad, CA, USA), and aliquots were stored at -20°C. Quantitative real-time TaqMan RT-PCR technology (7500Fast, Applied Biosystems, Foster City, CA, USA) was used to determine the expression levels of the selected target genes. The cycling conditions included a denaturing step at 95°C for 10 min and 50 cycles of 95°C for 15 s and 60°C for 1 min. The following TaqMan probes were used in the Q-RT-PCR analysis: ACOX1, Hs01074241_m1; CPT1A, Hs00912671_m1; CPT1B, Hs03046298_s1; ACADM, Hs00936580_m1 (Applied Biosystems, Foster City, CA, USA).

**SDS-PAGE**

Each differentiated adipocytes (0, 3, 7 days) were lysed in RIPA cell lysis buffer containing a protease inhibitor and phosphatase inhibitor cocktail (Sigma-Aldrich, St Louis, MO, USA). The lysate was then subjected to centrifugation at 15,000×*g* for 20 min, and the supernatant was used for the analysis. The protein concentration was determined using the Bradford method with bovine serum albumin as the standard. The proteins (40 µg/well) were loaded and fractionated using SDS-PAGE, and extracted in 7 parts for Nano LC-MS/MS analysis

**Mass spectrometric analysis (LTQ methods)**

Nano LC-MS/MS analysis was performed on an Agilent 1100 Series nano-LC and LTQ mass spectrometer (Finnigan^TM^ LTQ^TM^) (Thermo Electron, San Jose, CA). The capillary column used for LC-MS/MS (150 mm x 0.075 mm) was obtained from Proxeon (Odense M, city, Denmark) and slurry packed in house with 5 μm Magic C18 stationary phase (Michrom Bioresources, Auburn, CA, USA). The mobile phase A for the LC separation was 0.1% formic acid in deionized water, and the mobile phase B was 0.1% formic acid in acetonitrile. The chromatography gradient was set up to produce a linear increase from 5% B to 35% B in 70 min, from 70% B to 95% B in 10 min, and from 95% B to 5% B in 15 min. The flow rate was maintained at 600 nL/min after splitting. Mass spectra were acquired using data-dependent acquisition with full mass scan (350-1800 m/z) followed by MS/MS scans. Each MS/MS scan acquired was an average of one microscan on the LTQ. The temperature of the ion transfer tube was controlled at 200°C and the spray was 1.5-2.0 kV. The normalized collision energy was set at 35% for MS/MS.

**Figure S1. Time course of 590 nm light irradiation on lipolysis in differentiated adipocytes.**

Differentiated adipocytes were irradiated with 590 nm light at dose of 18 J/cm^2^ from 1 to 3 times (once a day, for 3 days). LDs in adipocytes were stained with Oil Red O. Scale bar = 200 μm.

**Figure S2. Effects of 590 nm light irradiation on FAS activity**

Differentiated adipocytes were irradiated with 590 nm visible light irradiation (18 J/cm^2^) 3 times (once a day, for 3 days). Then the cell lysates were collected and subjected to FAS activity assay. Based on the O.D. measured, the overall FAS enzyme activity was estimated by calculating the NADPH oxidation, using ε = 6220 M^-1^cm^-1^. The graphs depict the means ± S.D. of three independent experiments. N.S. = not significant (p>0.05).

**Figure S3.** **Effect of visible light irradiation on the gene expression involved in lipid metabolism.**

Using Q-RT-PCR analysis, the mRNA levels of *ACADM* (A), *ACOX1* (B), *CPT1A* (C), and *CPT1B* (D) were measured. Relative mRNA expression level was shown after once or 3 times exposure to forskolin or 457, 505, 530, 590, 660 nm wavelength visible light. The values represent the means ± S.E.M. of the mRNA expression corresponding to various genes normalized to human RPL13A expression.

**Figure S4.** **Photoreceptors are not detected in adipocytes.**

Each membrane fraction of adipocytes (0, 3, 7 days) was run on SDS-PAGE. Seven parts were extracted for LC-MS/MS analysis.

**Table S1. Analysis of perilipin 1 in 590 nm light irradiated adipocytes by next generation sequencing**

**Fig. S1**

**Fig. S2**

**Fig. S3**

**Table S1.**

| transcript | Gene_id | Gene_symbol | Description | Fold change | |
| --- | --- | --- | --- | --- | --- |
|  |  |  |  | 590 nm / control (1 time) | 590 nm / control  (3 times) |
| NM_002666 | 5346 | PLIN1 | perilipin 1 | -1.29 | 1.18 |
| NM_001145311 | 5346 | PLIN1 | perilipin 1 | -1.30 | -1.29 |

**Reference**

Kelley, D. S., G. J. Nelson, and J. E. Hunt. 1986. Effect of prior nutritional status on the activity of lipogenic enzymes in primary monolayer cultures of rat hepatocytes. *Biochem. J.* **235:** 87–90.
